# Relationship of Maternal 25-Hydroxyvitamin D₃ Deficiency With the Occurrence of Preterm Labor

**DOI:** 10.7759/cureus.108559

**Published:** 2026-05-09

**Authors:** Rubeena Badar, Muntiha Sarosh, Tehmina Zafar, Rupak Shrestha, Faiza Ghafoor, Anam Bashir, Tayyaba Majeed

**Affiliations:** 1 Obstetrics and Gynecology, Central Park Medical College, Lahore, PAK; 2 Obstetrics and Gynecology, Avicenna Medical College, Lahore, PAK; 3 Surgery, Hulhumale Hospital, Hulhumale, MDV; 4 Obstetrics and Gynecology, Maternity and Children Hospital (MCH), Hafar Al-Batin, SAU; 5 Obstetrics and Gynecology, Fatima Memorial Hospital, Lahore, PAK

**Keywords:** 25-hydroxyvitamin d₃, maternal health, patients, preterm labor, vitamin d deficiency

## Abstract

Background: Preterm labor remains a major contributor to neonatal morbidity and mortality, particularly in regions where nutritional deficiencies are common.

Objective: This study assessed the relationship between maternal vitamin D₃ deficiency and the occurrence of preterm labor.

Methods: This cross-sectional analytical study was conducted at Central Park Teaching Hospital, Lahore, from December 2023 to December 2024, and included 316 pregnant women. Participants were categorized into a preterm labor group (n = 148) and a term delivery group (n = 168). Serum 25-OH-D₃ levels were measured using a chemiluminescent immunoassay and classified as deficient, insufficient, or sufficient.

Results: Vitamin D₃ deficiency was highly prevalent (67.1%) and significantly more common in women with preterm labor (80.4%) than those with term deliveries (55.4%) (p < 0.001). Mean serum vitamin D₃ levels were markedly lower in the preterm group (15.8 ± 5.9 ng/mL) compared with the term group (22.4 ± 7.1 ng/mL) (p < 0.001). Logistic regression demonstrated that vitamin D₃ deficiency remained an independent predictor of preterm labor (adjusted OR = 2.51, 95% CI: 1.62-3.89). Additional significant factors included hemoglobin <10 g/dL (aOR = 1.68), sun exposure <30 minutes/day (aOR = 1.74), antenatal visits <4 (aOR = 1.82), and previous preterm birth (aOR = 1.91).

Conclusions: Maternal vitamin D₃ deficiency is strongly associated with preterm labor and remains a significant independent predictor even after adjusting for clinical and demographic factors. Given its high prevalence and modifiable nature, routine screening and targeted vitamin D₃ supplementation may help reduce the burden of preterm birth.

## Introduction

The phenomenon of preterm birth is troubling from a global obstetric perspective, as it contributes significantly to neonatal complications, death, and long-term functional deficits. Preterm labor is defined as the presence of uterine contractions and cervical alterations prior to 37 completed weeks of gestation [1]. At a global level, it accounts for nearly 11% of all deliveries, and rates are even higher in the developing world. The fundamental causes of preterm labor remain poorly defined, and the advances in obstetric care have done little to alleviate the preterm birth problem [2]. There remains a need to identify early, actionable, and modifiable risk factors to address this problem [3]. For its role in regulating calcium and bone metabolism, the vitamin D hormone has always been in the limelight. However, over the years, due to its various functions, considerable interest has been directed towards its active metabolite, 25-hydroxyvitamin D₃. Recent studies indicate that during pregnancy, vitamin D plays a significant role in placental development, immune modulation, inflammation, and myometrial relaxation and contraction [4].

Recent studies have confirmed a link between 25-hydroxyvitamin D₃ deficiency and gestational diabetes, preeclampsia, intrauterine growth restriction, and preterm birth. Because of vitamin D's modulating influence on the secretion of certain inflammatory cytokines and on smooth muscle contraction, a plausible and reasonable biological linkage between the deficiency and preterm activation of the labor pathway could be established [5]. Reports about the research on vitamin D point to the fact that such a deficiency on the part of the mother affects over 4-60% of the world’s pregnant women and seems to stem from the increased risk of spontaneous premature labor [6]. Overall, a lack of the vitamin yields increased and unregulated risks for inflammation, maternal infections, impaired placental implantation, and the loss of immune tolerance for the sustenance of pregnancy. Preterm risks are directed toward poverty-stricken Africans, the young, the obese, and the vitamin D-deficient African American race [7].

Deficient vitamin D levels correlate with low levels of that vitamin in the newborn, which, in turn, is a function of the mother’s vitamin D levels. Deficient maternal vitamin D levels are common, and so many newborns are not at risk but are at risk of insufficient and deficient 25(OH) vitamin D [8]. Recent research has unearthed low vitamin D as a contributor to the levels of health risk that are not directly associated with the traditional low vitamin D health risks of calcium deficiency and bone health [9]. New research is connecting maternal 25-hydroxyvitamin D₃ deficiency to negative obstetric outcomes and preterm labor, establishing one of the most frequently explored associations. Vitamin D deficiency may cause unregulated inflammatory responses, leading to premature activation of labor pathways because of the inflammatory cytokine dysregulation of IL-6, TNF-α, and IL-10, which are crucial in triggering the parturition cascade [10]. Furthermore, vitamin D deficiency contributes to the increased risk of genitourinary infections, which are known to cause preterm labor. Finally, a vitamin D deficiency may harm a woman’s ability to tolerate the immune system of a fetus and reinforce the angiogenesis of a placenta, contributing to the conditions that predispose pregnant women to early uterine contractions and cervical ripening [11]. 

Current literature still lacks strong local evidence on whether maternal 25-hydroxyvitamin D₃ deficiency is an independent predictor of preterm labor after adjusting for confounders like anemia, sun exposure, antenatal care, and previous preterm birth, especially in Pakistani populations.

This study aimed to determine the association between maternal serum 25-hydroxyvitamin D₃ deficiency and preterm labor and to evaluate whether vitamin D deficiency independently predicts preterm labor after adjustment for relevant maternal and obstetric factors.

## Materials and methods

This cross-sectional analytical study was conducted at Central Park Teaching Hospital, Lahore, from December 2023 to December 2024. Institutional Ethical and Review Board of Central Park Teaching Hospital, Lahore issued approval CPMC/IRB-No/1502. A total of 316 pregnant women were enrolled. Non-probability consecutive sampling methodology was utilized. All eligible women reporting to the facility during the study period were offered the opportunity to participate until the targeted sample size was achieved. Invited were women aged 18-40 years who were pregnant, had a singleton gestation, and were willing to have a serum 25-hydroxyvitamin D₃ (vitamin D) level drawn. Eligible were women who presented with spontaneous preterm labor (gestational age <37 weeks) or were admitted for term delivery (≥37 weeks). Women were excluded if they had a chronic illness, such as organ (kidney or liver) disease; malabsorption syndromes; autoimmune or pre-existing endocrine disorders; or those who were already on a high-dose vitamin D regimen. Women were also excluded if they had a twin/multiples pregnancy, if they had had an induced preterm delivery for maternal or fetal indications, or if they had incomplete data. Gestational age was determined primarily by first-trimester ultrasonography, where available. In women without an early ultrasound record, gestational age was estimated from the last menstrual period (LMP) only when the menstrual history was considered reliable, including patient certainty of dates and regular menstrual cycles. When both LMP- and ultrasound-based estimates were available, ultrasound dating was considered definitive if the discrepancy exceeded standard obstetric limits; cases with unresolved or clinically significant discordance were excluded to minimize gestational age misclassification.

Data collection procedure

After obtaining informed consent, participants underwent a structured interview to record demographic details, obstetric history, sun-exposure habits, dietary intake, and relevant clinical factors. Information was recorded regarding maternal age, parity, body mass index, socioeconomic status, obstetric history, antenatal care, hemoglobin level, smoking or passive smoking exposure, sun-exposure habits, and dietary vitamin D intake (Appendix 1). To reduce recall bias, exposure-related information was collected using predefined categories rather than open-ended questions. Sun exposure was assessed as the average daily duration of direct sunlight exposure to uncovered body areas over the preceding four weeks and was categorized as less than 30 minutes per day or 30 minutes per day or more. Dietary vitamin D intake was assessed according to the reported frequency of consumption of vitamin D-rich foods, including milk, eggs, fish, and fortified products, and was categorized as adequate when intake was reported at least three times per week and inadequate when it was reported less than three times per week. Although these measures were taken to reduce recall error, some degree of recall bias may still have remained because these variables were self-reported.

Blood samples for serum 25-hydroxyvitamin D₃ estimation were collected at the time of hospital presentation in both groups. In the preterm labor group, blood was drawn at the time of presentation with spontaneous preterm labor, whereas in the term delivery group, blood was collected at the time of admission for term delivery. All samples were analyzed in the same institutional laboratory to reduce inter-assay variation. Serum 25-hydroxyvitamin D₃ levels were measured using a commercially available chemiluminescent immunoassay kit from Abbott Laboratories. The assay had an analytical measurement range of 3.4 to 155.9 ng/mL, a lower detection limit of 3.4 ng/mL, and a functional sensitivity of 4.0 ng/mL. The intra-assay coefficient of variation ranged from 2.3% to 5.1%, while the inter-assay coefficient of variation ranged from 3.1% to 6.7%, indicating acceptable precision and reproducibility. All laboratory analyses were carried out under the same internal quality control procedures. Serum 25-hydroxyvitamin D₃ levels were classified according to standard clinical cut-offs as deficient if less than 20 ng/mL, insufficient if 20 to 30 ng/mL, and sufficient if greater than 30 ng/mL. The primary outcome of the study was the occurrence of preterm labor. Preterm labor was diagnosed clinically by the presence of regular uterine contractions with associated cervical changes before 37 completed weeks of gestation. The primary exposure variable was maternal serum 25-hydroxyvitamin D₃ level. Other independent variables evaluated included maternal age, parity, body mass index, socioeconomic status, antenatal care coverage, hemoglobin level, sun exposure, dietary vitamin D intake, previous preterm birth, interpregnancy interval, gestational diabetes, pregnancy-induced hypertension, urinary tract infection, and smoking or passive smoking exposure.

Data analysis

Data were entered and analyzed using IBM SPSS Statistics for Windows, Version 26 (Released 2018; IBM Corp., Armonk, New York, United States). Quantitative variables were summarized as mean ± standard deviation, while categorical variables were presented as frequencies and percentages. Baseline maternal characteristics were also compared between groups using the independent samples t-test for continuous variables and the chi-square test for categorical variables. A multivariate logistic regression model was then constructed to identify independent predictors of preterm labor. Variables entered into the regression model included vitamin D deficiency category, maternal age >30 years, BMI ≥30 kg/m², parity ≥3, hemoglobin <10 g/dL, sun exposure <30 minutes/day, antenatal visits <4, and previous preterm birth, as these were clinically relevant covariates and/or showed potential association with the outcome in initial analyses. A p-value of <0.05 was considered statistically significant.

## Results

Data were collected from 316 patients; the mean maternal age was similar between the preterm labor group (27.6 ± 4.9 years) and the term delivery group (28.1 ± 4.7 years). Most women in both groups were 25-30 years old, though the preterm group had a slightly higher proportion of younger women aged 18-24 years (40.5% vs. 34.5%). BMI values were comparable across groups, with a mean of 26.1 ± 4.0 kg/m² in preterm cases and 26.7 ± 3.8 kg/m² in term deliveries. Socioeconomic differences were modest, although a lower socioeconomic status appeared more common among the preterm group (58.1% vs. 49.4%) (Table 1).

**Table 1:** Baseline maternal characteristics (N = 316) Baseline characteristics are presented descriptively, and no formal statistical comparisons were performed, as these variables were not considered outcomes of interest. BMI: body mass index

Variable	Total (N = 316)	Preterm Labor (n = 148)	Term Delivery (n = 168)
Maternal age (years), mean ± SD	27.9 ± 4.8	27.6 ± 4.9	28.1 ± 4.7
Maternal age groups			
18–24 years	118 (37.3%)	60 (40.5%)	58 (34.5%)
25–30 years	129 (40.8%)	56 (37.8%)	73 (43.5%)
>30 years	69 (21.8%)	32 (21.6%)	37 (22.0%)
BMI (kg/m²), mean ± SD	26.4 ± 3.9	26.1 ± 4.0	26.7 ± 3.8
Parity			
Nulliparous	104 (32.9%)	51 (34.5%)	53 (31.5%)
Para 1–2	162 (51.3%)	72 (48.6%)	90 (53.6%)
Para ≥3	50 (15.8%)	25 (16.9%)	25 (14.9%)
Socioeconomic status			
Lower	169 (53.5%)	86 (58.1%)	83 (49.4%)
Middle	109 (34.5%)	46 (31.1%)	63 (37.5%)
Upper	38 (12.0%)	16 (10.8%)	22 (13.1%)
Antenatal care ≥4 visits	182 (57.6%)	74 (50.0%)	108 (64.3%)

Women with preterm labor had significantly lower mean serum vitamin D₃ levels (15.8 ± 5.9 ng/mL) than those with term deliveries (22.4 ± 7.1 ng/mL), with a clear statistical difference (p < 0.001). Vitamin D deficiency was much more frequent in the preterm group (80.4%) compared to the term group (55.4%), while sufficient levels were rare among preterm cases (4.7% vs. 19.6%). Sun exposure patterns followed a similar trend: 73.0% of preterm patients had <30 minutes/day exposure compared with 57.1% of term patients, indicating a lifestyle-related impact (Table 2).

**Table 2:** Vitamin D₃ levels and deficiency status

Variable	Preterm Labor (n = 148)	Term Delivery (n = 168)	Test Statistic	p-value
Mean serum 25-OH vitamin D₃(ng/mL), mean ± SD	15.8 ± 5.9	22.4 ± 7.1	t = −8.76	<0.001
Vitamin D₃ status			χ² = 22.45	<0.001
Deficient (<20 ng/mL)	119 (80.4%)	93 (55.4%)		
Insufficient (20–30 ng/mL)	22 (14.9%)	42 (25.0%)		
Sufficient (>30 ng/mL)	7 (4.7%)	33 (19.6%)		
Sun exposure <30 min/day	108 (73.0%)	96 (57.1%)	χ² = 8.30	0.004
Adequate dietary vitamin D intake	32 (21.6%)	60 (35.7%)	χ² = 7.65	0.006

Vitamin D deficiency showed a significant association with preterm labor, with a crude odds ratio of 2.90 (95% CI: 1.80-4.68, p < 0.001), indicating nearly threefold higher odds of preterm labor among deficient women. In contrast, insufficient vitamin D₃ levels were observed in 22 (14.9%) preterm labor patients and 42 (25.0%) term delivery patients, and did not show a statistically significant association with preterm labor (OR = 0.78, 95% CI: 0.43-1.39, p = 0.31). Sufficient vitamin D₃ levels were relatively uncommon in the preterm labor group, found in only seven (4.7%) patients compared with 33 (19.6%) patients in the term delivery group (Table 3).

**Table 3:** Association between vitamin D₃ status and preterm labor OR: odds ratio; CI: confidence interval; SD: standard deviation

Vitamin D₃ Category	Preterm Labor (n = 148)	Term Delivery (n = 168)	Crude OR (95% CI)	p-value
Deficient	119 (80.4%)	93 (55.4%)	2.90 (1.80–4.68)	<0.001
Insufficient	22 (14.9%)	42 (25.0%)	0.78 (0.43–1.39)	0.31
Sufficient	7 (4.7%)	33 (19.6%)	Reference	—

A history of previous preterm birth was higher in the preterm group (16.9% vs 9.5%), showing a significant recurrence pattern. Low hemoglobin levels (<10 g/dL) were also more frequent among preterm cases (39.9% vs 25.6%), and mean hemoglobin was slightly lower as well (10.5 ± 1.4 g/dL vs 10.8 ± 1.2 g/dL). Smoking or passive smoke exposure was more common in the preterm group (33.1% vs 23.2%) (Table 4).

**Table 4:** Obstetric and clinical factors associated with preterm labor

Variable	Preterm Labor (n = 148)	Term Delivery (n = 168)	Test Statistic	p-value
Previous preterm birth	25 (16.9%)	16 (9.5%)	χ² = 4.17	0.04
History of miscarriage	31 (20.9%)	27 (16.1%)	χ² = 1.21	0.27
Interpregnancy interval <18 months	41 (27.7%)	35 (20.8%)	χ² = 2.07	0.15
Gestational diabetes	19 (12.8%)	17 (10.1%)	χ² = 0.48	0.49
Pregnancy-induced hypertension	16 (10.8%)	12 (7.1%)	χ² = 1.33	0.25
Hemoglobin (g/dL), mean ± SD	10.5 ± 1.4	10.8 ± 1.2	t = −2.17	0.03
Hemoglobin <10 g/dL	59 (39.9%)	43 (25.6%)	χ² = 7.05	0.008
Urinary tract infection	34 (23.0%)	29 (17.3%)	χ² = 1.56	0.21
Smoking/passive smoking	49 (33.1%)	39 (23.2%)	χ² = 3.91	0.048

Vitamin D₃ deficiency was a significant independent predictor of preterm labor, with women having deficiency showing higher odds of preterm delivery (aOR = 2.51, 95% CI: 1.62-3.89, p < 0.001). Similarly, hemoglobin <10 g/dL (aOR = 1.68, 95% CI: 1.05-2.67, p = 0.03), sun exposure <30 minutes/day (aOR = 1.74, 95% CI: 1.14-2.65, p = 0.01), fewer than four antenatal visits (aOR = 1.82, 95% CI: 1.19-2.78, p = 0.006), and a history of previous preterm birth (aOR = 1.91, 95% CI: 1.04-3.49, p = 0.03) were also significantly associated with increased risk of preterm labor (Table 5).

**Table 5:** Multivariate logistic regression analysis predicting preterm labor BMI: body mass index; OR: odds ratio; CI: confidence interval; SD: standard deviation; aOR: adjusted odds ratio

Variable	aOR (95% CI)	p-value
Vitamin D₃ deficiency	2.51 (1.62–3.89)	<0.001
Insufficient vitamin D₃	0.81 (0.46–1.43)	0.31
Maternal age >30 years	1.12 (0.69–1.82)	0.64
BMI ≥30 kg/m²	1.27 (0.75–2.16)	0.38
Parity ≥3	1.19 (0.67–2.08)	0.55
Hemoglobin <10 g/dL	1.68 (1.05–2.67)	0.03
Sun exposure <30 minutes/day	1.74 (1.14–2.65)	0.01
Antenatal visits <4	1.82 (1.19–2.78)	0.006
Previous preterm birth	1.91 (1.04–3.49)	0.03

## Discussion

This study demonstrates an association and consistent relationship between maternal 25-hydroxyvitamin D₃ deficiency and the occurrence of preterm labor in a cohort of 316 pregnant women. The average serum vitamin D₃ level in women with preterm labor is also considerably lower (15.8 ± 5.9 ng/mL) than in those with term deliveries (22.4 ± 7.1 ng/mL), indicating a clear association between exposure and the severity of the outcome. The present study results are consistent with prior studies and show a high prevalence of preterm labor in women with lower vitamin D status. 

A notable finding of the present study was that vitamin D₃ insufficiency did not show a significant independent association with preterm labor, whereas overt deficiency remained significant after adjustment. This may suggest a threshold effect, in which only more severe depletion of maternal vitamin D₃ is sufficient to impair the immunologic, inflammatory, and placental mechanisms involved in maintaining pregnancy. A deficiency of vitamin D, coupled with unregulated cytokine changes, poor placental development, and an increased risk of intrauterine infection, may accelerate uterine contractility [12]. Our data support the existing evidence that women with less than 30 mins of sun exposure (insufficient) are more likely to have preterm labor (73.0% vs. 57.1%), and logistic regression corroborated that less exposure to sunlight increased the odds of preterm birth (aOR = 1.74). This phenomenon is consistent with prior studies showing that vitamin D restriction due to environmental and behavioral factors increases obstetric risks. 

The multivariate model, after controlling for maternal age, BMI, parity, socioeconomic status, frequency of antenatal care, and anemia, shows that vitamin D deficiency remains an independent risk factor for preterm labor (aOR = 2.51). This demonstrates that the association is neither a by-product of prevailing socioeconomic status nor of general health problems but rather emphasizes a specific biological risk that may be tied to vitamin D deficiency [13]. The literature is replete with similarly reported adjusted odds ratios that demonstrate the association with high consistency and reliability across different geographical and cultural settings. The obstetric factors within our sample also had significant associations. The preterm group had a higher proportion of individuals with a history of prior preterm birth (16.9%), and this history independently predicted recurrence (aOR = 1.91). Prevalence of maternal anemia (<10 g/dL) was higher among preterm cases (39.9% vs. 25.6%) and remained significant in the model (aOR = 1.68). 

The association between anemia, previous obstetric outcomes, and the risk of preterm delivery is well documented, and, after accounting for these clinical contributors, the deficiency of vitamin D₃ had the greatest impact in the model [14,15]. This indicates that vitamin D₃ deficiency is a potentially modifiable clinical risk factor. The effect of the quantity of antenatal care received is also noteworthy. For women with fewer than four antenatal visits, the odds of experiencing preterm labor were greater (aOR = 1.82), which echoes previous studies highlighting the significance of antenatal care as a vital monitoring tool. Additional antenatal care restrictions would likely intensify the effects of vitamin D deficiency, as more vital assessments and nutrient supplements would be missed [16]. The combination of an unmonitored nutritional deficiency, unmeasured and unmonitored social metrics, and a lack of antenatal care places women in an environment of increasing risk for preterm labor [17]. 

A notable finding in the present study was that only overt vitamin D₃ deficiency, and not insufficiency, remained significantly associated with preterm labor after adjustment for confounding variables. Women with deficient vitamin D₃ levels had significantly increased odds of preterm labor, whereas the insufficient category did not show a statistically significant association. This may indicate a possible threshold effect, whereby the risk of preterm labor becomes clinically meaningful only below a certain serum vitamin D₃ level. From a clinical perspective, this suggests that severe deficiency may be more relevant than borderline insufficiency when assessing preterm labor risk. When analyzing study results, multiple factors must be understood as limitations. The inability to establish a cause-and-effect relationship between preterm labor and vitamin D deficiency, given the cross-sectional nature of the study, is the first such limitation. 

This study has several limitations. First, because of its cross-sectional design, a causal relationship between maternal vitamin D₃ deficiency and preterm labor cannot be established. Second, selection bias may have been present because participants were recruited through non-probability consecutive sampling from a single tertiary care hospital. Third, recall bias may have affected self-reported variables such as sun exposure and dietary vitamin D intake, despite the use of a structured questionnaire and predefined recall period. Fourth, seasonal variation in vitamin D levels was not specifically analyzed, although the study was conducted over a one-year period; this may have influenced serum vitamin D status through changes in sunlight exposure across seasons. An additional limitation of the present study is that seasonal variation in vitamin D status was not analyzed separately. Because the study was conducted over a one-year period from December 2023 to December 2024, fluctuations in sunlight exposure across different seasons may have influenced maternal serum 25-hydroxyvitamin D₃ levels and possibly the observed distribution of deficiency between groups. Since sun exposure was one of the variables assessed in this study, the absence of seasonal stratification should be considered when interpreting the findings. Finally, vitamin D₃ was measured only once at the time of presentation/admission, which may not fully reflect maternal vitamin D status throughout pregnancy.

## Conclusions

Maternal 25-hydroxyvitamin D₃ deficiency was significantly associated with preterm labor in this study population. Women with preterm labor had lower mean serum vitamin D₃ levels and a higher frequency of overt deficiency than women who delivered at term. After adjustment for relevant clinical and demographic factors, vitamin D₃ deficiency remained an independent predictor of preterm labor, whereas vitamin D₃ insufficiency did not show a significant association. These findings suggest that overt maternal vitamin D₃ deficiency may be an important and potentially modifiable risk factor for preterm labor. However, given the cross-sectional design, single-center setting, and potential influence of residual confounding and seasonal variation, the results should be interpreted with caution.

## Figures and Tables

**Figure 1:**
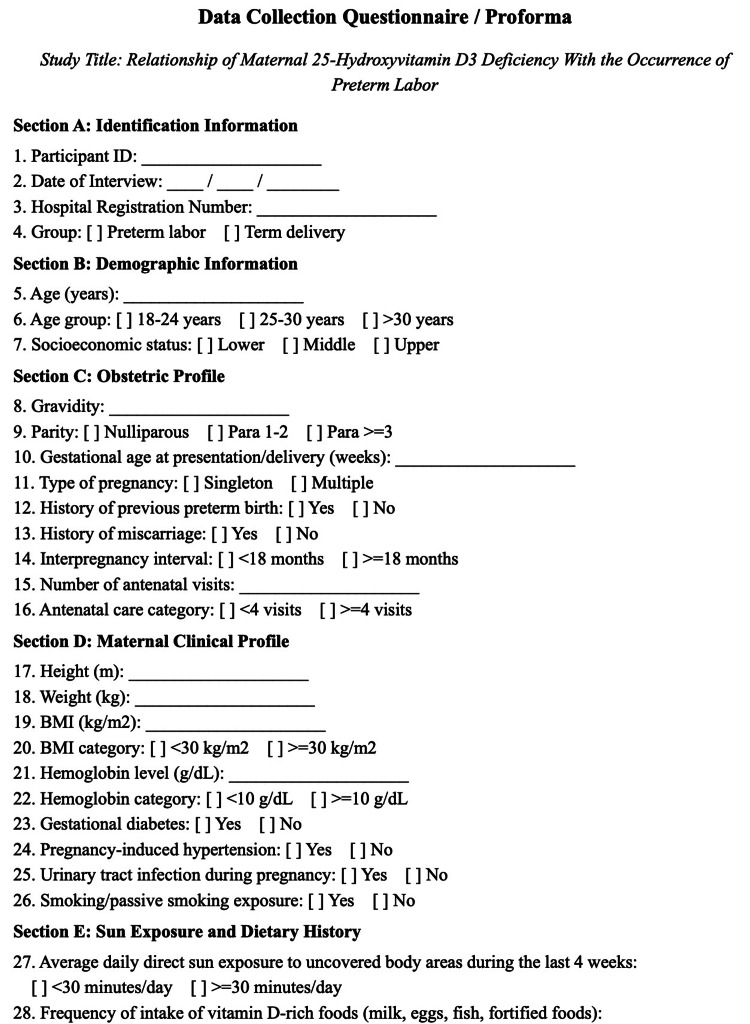
Data collection questionnaire (page 1)

**Figure 2:**
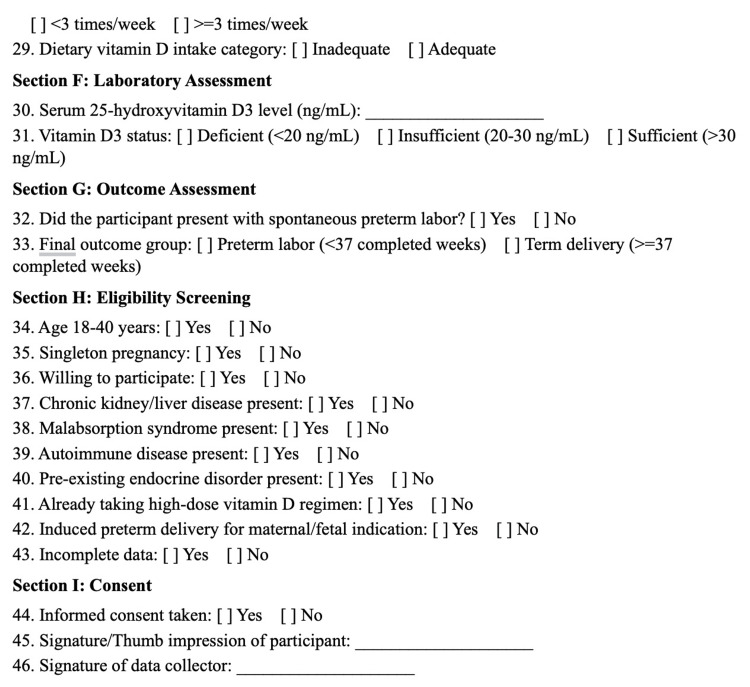
Data collection questionnaire (page 2)

## Appendices

Appendix 1
